# Nanoscale Zero-Valent Iron Decorated on Bentonite/Graphene Oxide for Removal of Copper Ions from Aqueous Solution

**DOI:** 10.3390/ma11060945

**Published:** 2018-06-04

**Authors:** Jicheng Shao, Xiaoniu Yu, Min Zhou, Xiaoqing Cai, Chuang Yu

**Affiliations:** 1 College of Civil Engineering and Architecture, Wenzhou University, Wenzhou 325035, China; shaojicheng@aliyun.com (J.S.); xnyu@wzu.edu.cn (X.Y.); zhoumin480855165@aliyun.com (M.Z.); geoyuchuang@163.com (C.Y.); 2 College of Chemistry and Materials Engineering, Wenzhou University, Wenzhou 325035, China

**Keywords:** zero-valent iron, bentonite, graphene oxide, copper ions, adsorption

## Abstract

The removal efficiency of Cu(II) in aqueous solution by bentonite, graphene oxide (GO), and nanoscale iron decorated on bentonite (B-nZVI) and nanoscale iron decorated on bentonite/graphene oxide (GO-B-nZVI) was investigated. The results indicated that GO-B-nZVI had the best removal efficiency in different experimental environments (with time, pH, concentration of copper ions, and temperature). For 16 hours, the removal efficiency of copper ions was 82% in GO-B-nZVI, however, it was 71% in B-nZVI, 26% in bentonite, and 18% in GO. Bentonite, GO, B-nZVI, and GO-B-nZVI showed an increased removal efficiency of copper ions with the increase of pH under a certain pH range. The removal efficiency of copper ions by GO-B-nZVI first increased and then fluctuated slightly with the increase of temperature, while B-nZVI and bentonite increased and GO decreased slightly with the increase of temperature. Lorentz-Transmission Electron Microscope (TEM) images showed the nZVI particles of GO-B-nZVI dispersed evenly with diameters ranging from 10 to 86.93 nm. Scanning electron microscope (SEM) images indicated that the nanoscale iron particles were dispersed evenly on bentonite and GO with no obvious agglomeration. The q_e,cal_ (73.37 mg·g^−1^ and 83.89 mg·g^−1^) was closer to the experimental value q_e,exp_ according to the pseudo-second-order kinetic model. The q_m_ of B-nZVI and GO-B-nZVI were 130.7 mg·g^−1^ and 184.5 mg·g^−1^ according to the Langmuir model.

## 1. Introduction

The rapid development of industry has exerted an influence on the ecological environment, with a great deal of land and water resources being polluted by human production. Among these pollutions, metal ions occupy a large part. These toxic and harmful substances have brought various problems to the ecological environment and human health, seriously threatening biological health. The pollution of Cu(II) has become a serious problem in land and water resources. More attention has been paid to the pollution of Cu(II). When Cu(II) accumulates to a certain amount, the victims will have symptoms of diseases including physiological obstruction, growth stagnation, or even death [1]. Many methods can be adopted to remove heavy metal ions from polluted water including ions exchange, chemical precipitation, biosorption, infiltration, electrolysis, membrane separation, mineral adsorption, and so on [2,3,4,5,6,7]. However, these methods also have the disadvantages of a long treatment cycle, high cost, and are only suitable for the treatment of low concentrations of metal ion contaminated wastewater. A large number of studies on nanoscale zero-valent iron (nZVI) have been carried out to remove metal ions in recent years. Compared with the methods mentioned above, nZVI has many advantages including faster and more complete reactions, it is suitable for remediation and degradation of various kinds of metal ions, and has better injectability into aquifers for the in situ remediation of soil and groundwater [8]. The nZVI can remove hexavalent(VI), lead(II), arsenic(III), etc. well, and the removal efficiency is very high for its outstanding characteristics like large specific surface area, high reactivity, and reduction capability [9,10,11,12]. However, nZVI is similar to other nanomaterials, as an ultra-fine powder with a particle size less than 100 nm, it has a strong tendency to agglomerate into larger particles and is easily oxidized in air or can even combust spontaneously, which results in an adverse effect on both effective surface area and reactive activity [13,14]. The adverse effects are caused by Van der Waals and magnetic forces when synthesized materials are in solution, which can be diminished through the immobilization of the nZVI on the surface or inside different materials such as silica, activated carbon, starch, polymers, and so on [15,16].

Graphene oxide (GO) and bentonite were used to support nZVI in the study. Bentonite with a layer structure has a large specific surface area (32.4 m^2^·g^−1^) that can offer supporting sites for nZVI particles, preventing the agglomeration of nZVI particles [17]. Bentonite is mainly composed of montmorillonite. The montmorillonite interlayer structure is composed of two layers of silicon tetrahedron lamellae sandwiched by a layer of aluminum (magnesium) oxygen octahedral lamellar lamella 2:1 layered silicate. Montmorillonite unit layers have enough space to support zero-valent iron particles. Bentonite can swell and has a uniform dispersion in water, which is a nice carrier. Bentonite has excellent adsorption capacity, ion exchange properties, and many other merits that make bentonite considered to have a great application potential in metal ions wastewater treatment. Low price, being environmentally-friendly, and so on make it to be an ideal material to support nZVI. Graphene is a new type of 2D flaky material with a single layer thickness of only 0.3354 nm and large specific surface area (2630 m^2^·g^−1^), but graphene is neither hydrophilic nor lipophilic. Graphene oxide also has a large surface area and flaky structure, and graphene is different, graphene oxide surface with a large number of active functional groups, such as hydroxyl, carboxyl and so on, which greatly improves its compatibility with the polymer. Graphene oxide has excellent hydrophilicity and uniformly dispersed in water under the action of ultrasound. Graphene oxide has strong adsorption properties and flake structure, can be utilized for the removal of heavy metal ions and support other substances. Compared to graphene, graphene oxide has a large specific surface area and flaky structure that can be utilized to support other substances including nZVI [18,19]. Graphene oxide can be used as an additive to improve the removal efficiency of heavy metal ions. Therefore, graphene oxide (GO) and bentonite were used to support nZVI in this study.

## 2. Materials and Methods

### 2.1. Materials

Graphene oxide (layer < 3, purity 99%) was purchased from J&K Scientific LTD (Shanghai, China). Bentonite was taken from Chunyuan Company (Jinzhou, China) and chemical composition of bentonite was shown in Table 1. Ferric chloride tetrahydrate, sodium borohydride, anhydrous ethanol, sodium hydroxide and copper nitrate were purchased from Sinopharm Chemical Reagent Co., Ltd (Beijing, China) (analytical purity).

### 2.2. Preparation of Graphene Oxide/Bentonite Decorated Nano-Iron (GO-B-nZVI)

A quantity of 0.2 g of graphene oxide and 300 mL of high purity water were added into a beaker for 1 h of ultrasonic vibration. Bentonite (1.4 g) was added to the above solution and stirred for 4 h. Then, 2.138 g of ferric chloride tetrahydrate was added into the mixture solution of graphene oxide and bentonite for 1 h of ultrasonic vibration. Subsequently, 200 mL of absolute ethanol was added into the mixture solution of graphene oxide, bentonite, and ferric chloride tetrahydrate for 1 h of ultrasonic vibration. Next, 0.815 g of sodium borohydride (100 mL, pH = 11) was slowly added to the mixture solution of graphene oxide, bentonite, and ferric chloride tetrahydrate. The dropping process was about 15 min and was stirred for 20 min. The supernatant was decanted by centrifugation, and the remaining solid was washed three times with high purity water and absolute ethanol, respectively. The material was then lyophilized in a lyophilizer. Finally, the specimen was kept in the refrigerator (−85 °C) in a Ziploc bag, recorded as GO-B-nZVI. Under the same conditions, B-nZVI was synthesized without graphene oxide. Ultrasonic vibration and agitation can accelerate the dissolution and reaction of the materials. The preparation formula of nano-iron (GO-B-nZVI and B-nZVI) by liquid-phase reduction method was as follows [10]:

$${\mathrm{Fe}}^{2+}+{2B}_{4}^{-}+6H_{2}O\to 2\mathrm{Fe}(s)+2B{\left(OH\right)}_{3}+7H_{2}↑$$

### 2.3. Characterization of Materials

Specific surface area was measured by the BET-N_2_ method using a Micromeritics ASAP2020HD88 Chemisorption Surface Area Analyzer (Micromeritics Instrument Co., Ltd., Shanghai, China). X-ray diffraction analysis (XRD) was carried by a D8-Advance X diffraction meter (40 kV, 40 mA; Bruker Company, Karlsruhe, Germany) with Cu (λ = 1.5406 Å) irradiation at the rate of 0.15 s/step in the range of 5°–83°. The Fourier transform infrared spectroscopy (FTIR) of samples was determined by the TENSOR27 FTIR (Zhengzhou Great Wall Science and Industry Co., Ltd., Zhengzhou, China), and the scanning range was 4000–400 cm^−1^. Scanning electron microscope (SEM) images of the specimens were obtained on a JSM-6700F instrument (JOEL, Tokyo, Japan). Lorentz–Transmission Electron Microscope (TEM) images of the specimens were obtained on a JEM 2100F instrument (JOEL, Tokyo, Japan).

### 2.4. Removal Efficiency of Copper Ions

The Cu(II) solution was prepared by dissolving a certain amount of copper nitrate in ultrapure water. The concentration of the Cu(II) before and after adsorption was determined using an atomic absorption spectrometer (AAS, Shimadzu Company, Kyoto, Japan). The concentration of Cu(II) was calculated using Equation (1):

*Abs* = 0.12*Conc* + 0.015 (1)

where *Abs* is the absorbance value and *Conc* is the concentration of Cu(II) (mg·L^−1^).

An adsorbent dosage of 0.1 g L^−1^ (bentonite, GO, B-nZVI, and GO-B-nZVI) was dispersed in Cu(II) contaminated water using an ultrasound bath for 5 min and the solution was placed in the laboratory at constant temperature. At different time intervals, a certain amount of Cu(II) contaminated solution was taken and the concentration of Cu(II) was measured by an atomic absorption spectrometer. The pH of the solution was adjusted using 0.1 mol HNO_3_ or 0.1 mol NaOH. The removal efficiency of Cu(II) was calculated using Equation (2):

R = (1 − Ce/Co) × 100%(2)

where R is the removal efficiency of Cu(II); Ce is the concentration of Cu(II) (mg L^−1^) at different time intervals; and Co is the initial concentration of Cu(II) (mg L^−1^).

## 3. Results and Discussion

### 3.1. Composition and Microstructure of Synthesized Materials 

**Figure 1:**
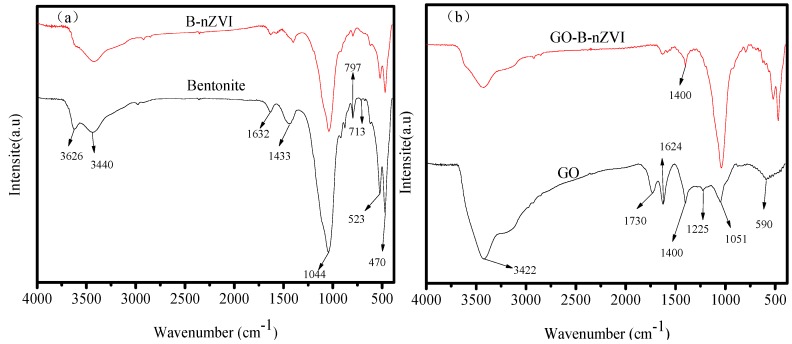
Fourier transform infrared spectroscopy (FTIR) spectra of bentonite, B-nZVI, GO, and GO-B-nZVI: (**a**) bentonite and B-nZVI, (**b**) GO and GO-B-nZVI.

The strong absorption peak near 1044, 523, and 470 cm^−1^ is the Si–O, Al–Si–O, and Si–O–Si bending vibration peak, respectively, as shown in Figure 1a [20]. The absorption peak near 797 cm^−1^ was caused by the antisymmetric stretching vibration of Si–O–Si in the bentonite. The absorption peak near 3626 cm^−1^ is the vibratory absorption peak of the hydroxyl group, and the absorption peak near 3430 cm^−1^ is the bending vibration peak of H–O–H for water, which is a reflection of the interlinear adsorption of water on bentonite. There was a vibrational absorption peak of O−H at 1632 cm^−1^, which was a reflection of crystal water in the bentonite lattice. Bentonite used in this research composition contained silicon dioxide, aluminum oxide, and water. Compared with bentonite, B-nZVI had a similar absorption peak. The synthesizing material process did not destroy the bentonite structure and chemical composition. Bentonite played a role in dispersing nZVI, which could be proved by the TEM and SEM images. Figure 1b shows the FTIR spectra of GO and GO-B-nZVI. The bond near 1051 cm^−1^ was most often related to the stretching vibration of C–O. The peak at 1220, 1400, and 1730 cm^−1^ ascribed to the stretching vibration of C–O–C, stretching vibration of C–O, and stretching vibration of C=O, respectively [21]. The peak at 1624 cm^−1^ might correspond to the stretching vibration of the C=C [22,23]. These peaks belong to the inherent peaks of graphene oxide, which could be found in similar literature. With just 0.1 g of GO added during the process of synthesizing the material, the stretching vibration of C–O at 1400 cm^−1^ could be found in GO-B-nZVI. Other peaks belonging to bentonite could also be found in GO-B-nZVI, which indicated that the three kinds of raw materials were well combined.

**Figure 2:**
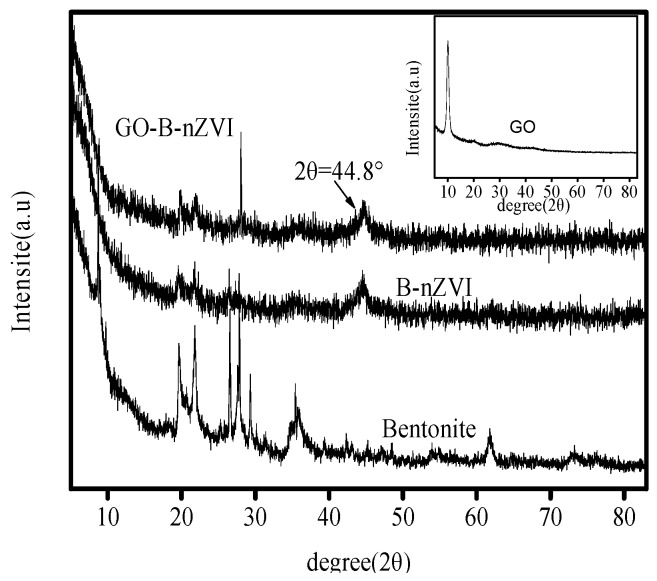
X-ray diffraction analysis (XRD) patterns of bentonite, B-nZVI, GO-B-nZVI, and GO.

X-ray diffraction patterns of bentonite, B-nZVI, GO and GO-B-nZVI are shown in Figure 2. The XRD of bentonite shows that the peak at 19.7° is sodium bentonite, and the bentonite is 2:1 of the mineral structure. The peaks at 21.9° and 26.6° represent quartz. The peak at 27.8° stands for the feldspar. The peak at 29.4° represents calcite. These compounds are in accordance with the chemical composition of Table 1. The characteristic (110) diffraction peak of iron at a 2-theta value was 44.8°, indicating the presence of zero-valent Fe in B-nZVI and GO-B-nZVI [24]. The phenomenon indicates that zero-valent iron was prepared successfully in the experiment.

The BET area of bentonite, GO, B-nZVI, and GO-B-nZVI are shown in Table 2. The BET area of B-nZVI was 42.88 m^2^·g^−1^, which was larger than that of bentonite whose BET area was 32.4 m^2^·g^−1^. Bentonite could reduce the agglomeration of nZVI and make the nZVI present a more dispersed state, thus increasing the specific surface area of the synthetic material [25,26]. Furthermore, the average pore diameter of bentonite was 10.57 nm, which was smaller than that of B-nZVI (11.56 nm), indicating that nZVI could expand the bentonite layer clearance. The BET area of GO-B-nZVI was 47.32 m^2^·g^−1^, indicating that GO could enlarge the specific surface area of B-nZVI. The nZVI showed a more dispersed state with the addition of GO, which can also be used to support nZVI [27].

**Figure 3:**
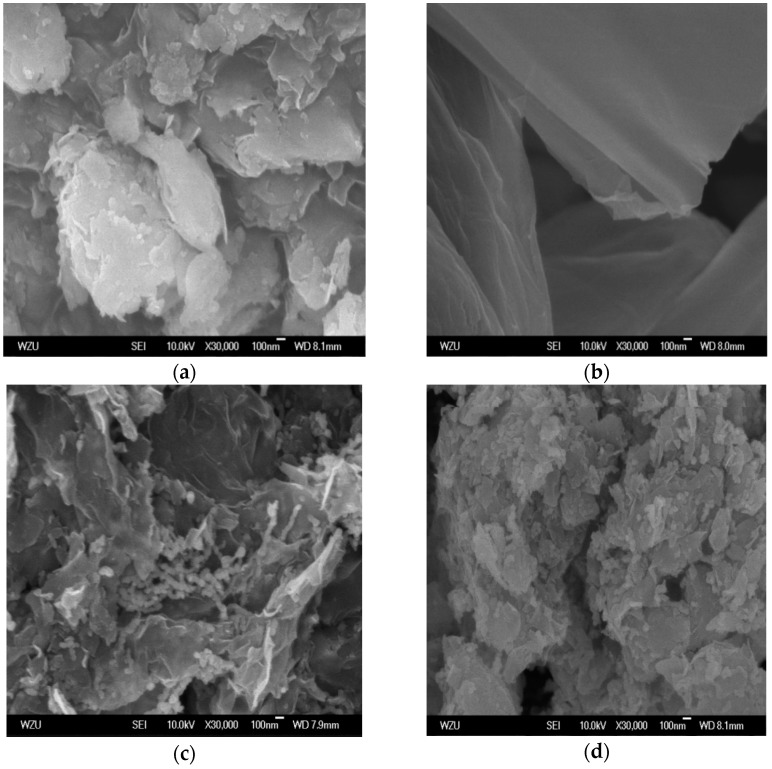
Scanning electron microscope (SEM) images of (**a**) bentonite, (**b**) GO, (**c**) B-nZVI, and (**d**) GO-B-nZVI.

The morphology of bentonite, GO, B-nZVI, and GO-B-nZVI can be observed in Figure 3. Bentonite presented a layer structure which could provide a good platform for nZVI, preventing the agglomeration of nZVI, as shown in Figure 3a. Figure 3c indicates that the iron particles dispersed in bentonite, with some chain-like iron particles and spherical iron particles attaching to the surface of bentonite, which has also been observed in other studies [28]. GO has a flaky structure with a large specific surface area, so could provide attaching sites for the iron particles as shown in Figure 3b. In Figure 3d, the iron particles decorated on the bentonite/graphene oxide were spherical and uniformly dispersed on the surface of the bentonite and graphene oxide, with no obvious agglomeration. GO enhanced the resistance against the particle aggregation through the electrostatic repulsion and steric hindrance. The sterically hindered effect was the main driving force that hindered the oxidation and agglomeration of nZVI [29].

**Figure 4:**
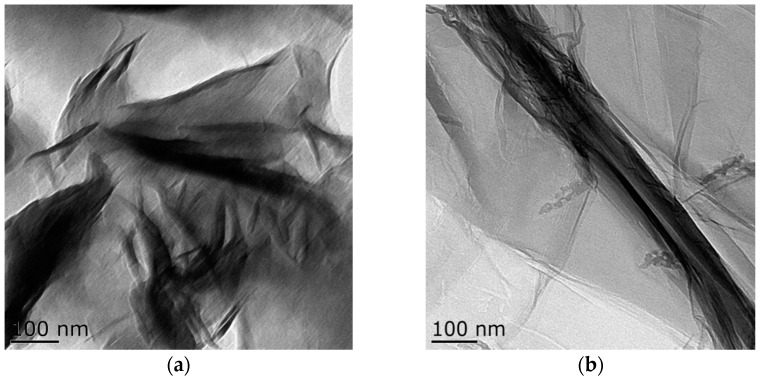

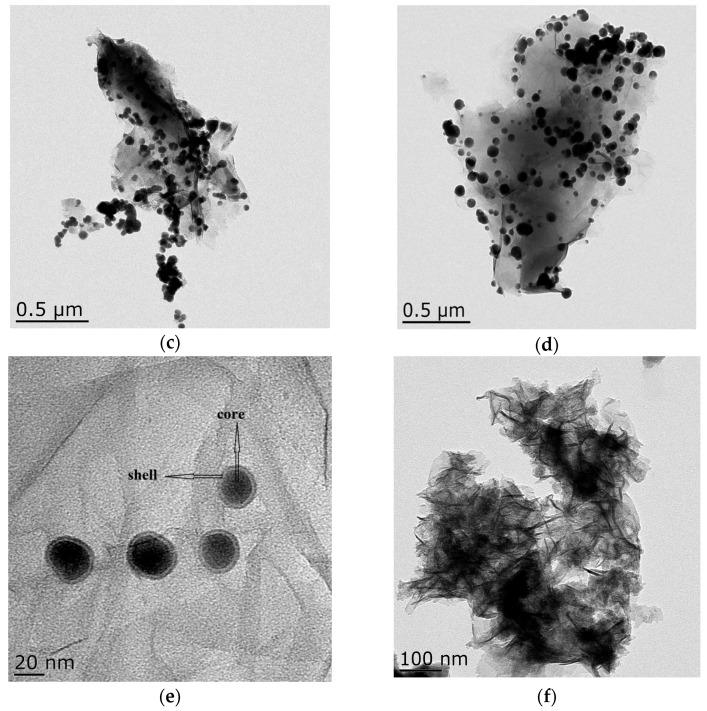
Lorentz-Transmission Electron Microscope (TEM) images of (**a**) bentonite, (**b**) GO, (**c**) B-nZVI, (**d**) GO-B-nZVI, (**e**) core–shell structure of nZVI, and (**f**) GO-B-nZVI after the removal of copper ions.

**Figure 5:**
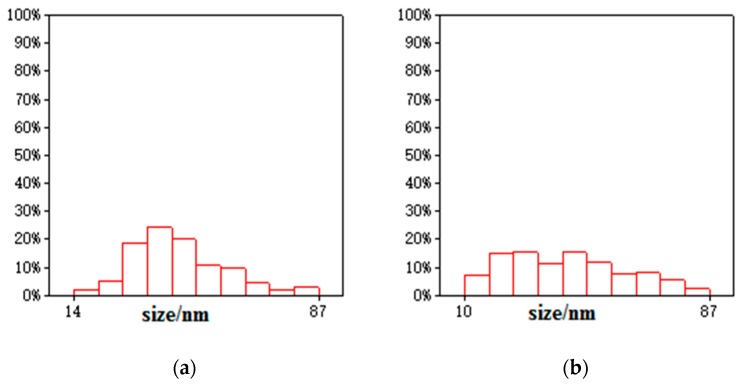

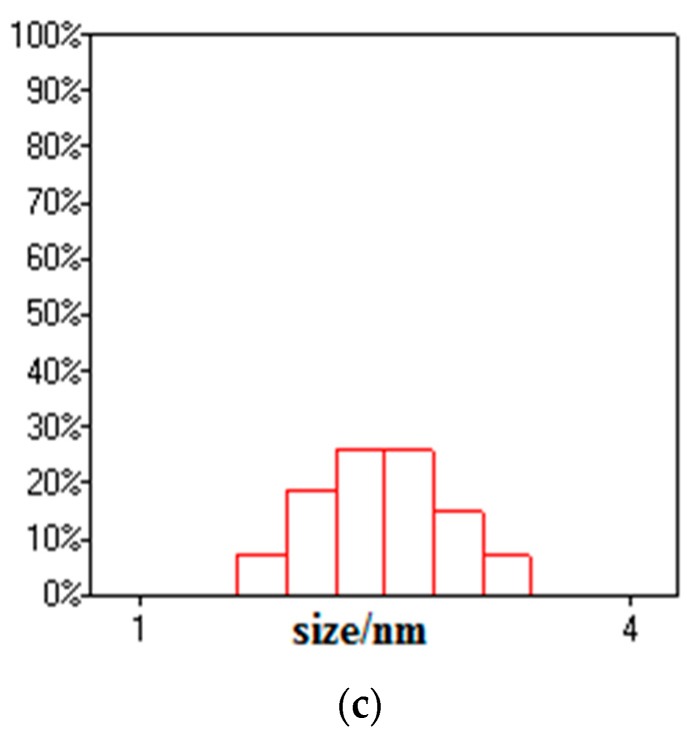
Diameter distribution of (**a**) B-nZVI, (**b**) GO-B-nZVI, and (**c**) core–shell structure of nZVI.

Transmission electron microscopy images of the samples are shown in Figure 4. Bentonite and GO showed a flake structure, which provided a good attaching platform for the iron particles. Figure 4c showed the TEM image of B-nZVI. The nZVI was dispersed uniformly on the surface of bentonite, and particle size of the nZVI was nanoscale. The TEM image of GO-B-nZVI and the nZVI was well dispersed, with diameters ranging from 10 nm to 86.93 nm and a mean value of 42.35 nm, as shown in Figure 4d and Figure 5b. From the TEM and SEM, the nZVI of GO-B-nZVI presented a more evenly distributed state than that of B-nZVI, with no agglomeration of iron particles and chain-like iron particles. Studies have reported that iron nanoparticles possess a core-shell structure, with the zero-valent Fe core surrounded by the oxidized part (shell), which can preserve the iron nanoparticles against further oxidation [30,31], as shown in Figure 4e. Results indicated that the shell thick was about 1.74–3.36 nm with average value of 2.58 nm (Figure 5c). Figure 4f shows the TEM image of GO-B-nZVI after the removal of copper ions. It was found that the iron particles disappeared with the copper ions, indicating that the iron particles would be consumed when the copper ions reacted with iron particles attached to the surface of bentonite and GO. It is likely that the removal of copper ions in solution by GO-B-nZVI depends largely on the reduction capability of nZVI (replacement reaction).

### 3.2. The Effect of Time, pH, Temperature, and Concentration of Copper Ions on Removal Efficiency

**Figure 6:**
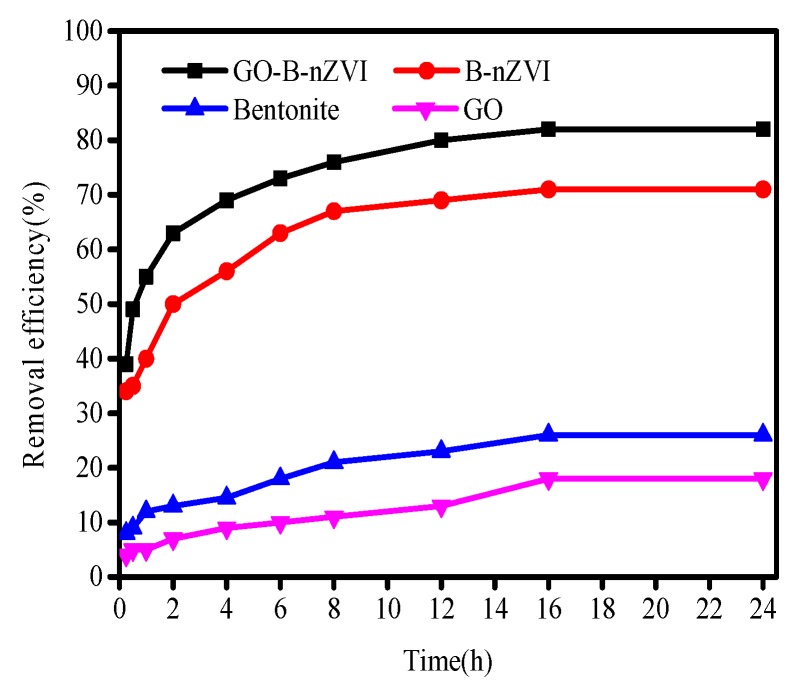
Removal efficiency of Cu(II) by bentonite, GO, B-nZVI, and GO-B-nZVI with time (Temperature = 18 °C; adsorbent dose = 1 g·L^−1^; pH = 5 ± 0.3; concentration of Cu(II) = 100 mg·L^−1^).

Figure 6 shows that the removal efficiency of Cu(II) by GO-B-nZVI was better than that of bentonite, graphene oxide, and B-nZVI, when pH, adsorbent, concentration of Cu(II), and temperature were 5 ± 0.3, 1 g·L^−1^, 100 mg·L^−1^, and 18 °C, respectively. After 16 h, the removal efficiency of GO-B-nZVI reached 82%, while the removal efficiency of Cu(II) by B-nZVI, bentonite, and GO were 71%, 26%, and 18%, respectively. Under the same experimental conditions, only B-nZVI and GO-B-nZVI had a high removal efficiency for Cu(II) in solution. The results indicated that iron nanoparticles played a leading role in the removal of Cu(II), however, bentonite and graphene oxide played a role in dispersing the nZVI particles. High removal efficiency of Cu(II) by B-nZVI was due to the iron particles with strong adsorbability to a large extent. Bentonite could prevent the agglomeration of iron particles during the process of synthesizing materials in aqueous solution. GO-B-nZVI had a higher removal efficiency of Cu(II) when compared with B-nZVI. This was most likely due to the reason that GO could increase the dispersion of iron particles [29].

**Figure 7:**
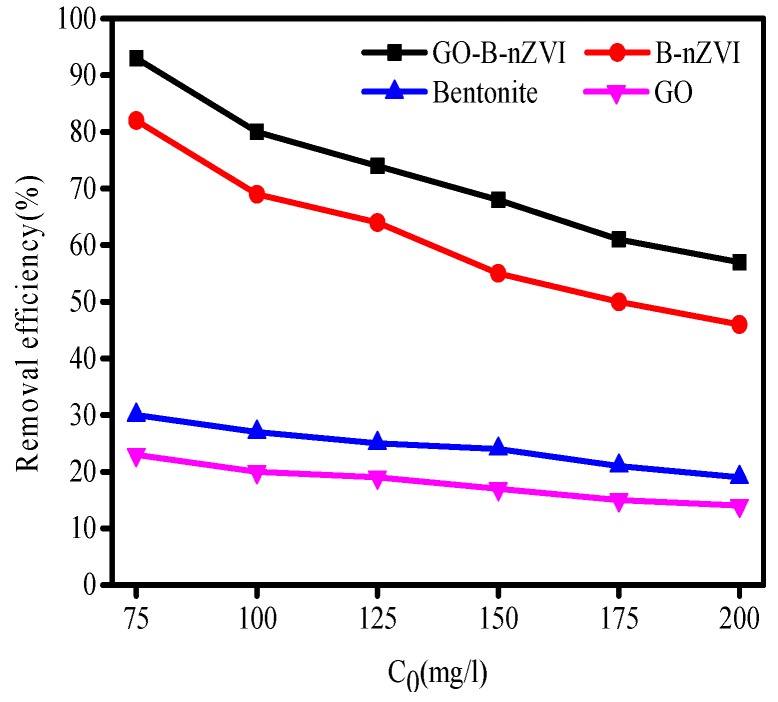
Removal efficiency of Cu(II) by bentonite, GO, B-nZVI, and GO-B-nZVI with concentration of Cu(II) (Temperature = 18 °C; adsorbent dose = 1 g·L^−1^; pH = 5 ± 0.3; adsorption time = 12 h).

The removal efficiency of Cu(II) by bentonite, GO, B-nZVI, and GO-B-nZVI with various concentrations are shown in Figure 7. The initial concentration of Cu(II) had a great influence on the removal efficiency by the above four materials, especially for B-nZVI and GO-B-nZVI. The removal efficiency of Cu(II) by the above four adsorbents decreased with the increase of the initial concentration. When the initial concentration of Cu(II) was 75 mg·L^−1^, the removal efficiency of Cu(II) by GO-B-nZVI was the best, which reached 93%, however, the initial concentration of Cu(II) was 200 mg·L^−1^, the removal efficiency of Cu(II) (57%) by GO-B-nZVI was lower than the other absorbents. This was due to the limited adsorption sites of the iron particles, and before the high concentrations of Cu(II) was adsorbed completely, the iron particles had reached a state of adsorption saturation [32,33]. The GO-B-nZVI showed better removal efficiency of Cu(II) than that of B-nZVI in the experiment. This phenomenon supports the point that nZVI with a smaller size, less agglomeration, had a higher removal efficiency of metal ions [34].

**Figure 8:**
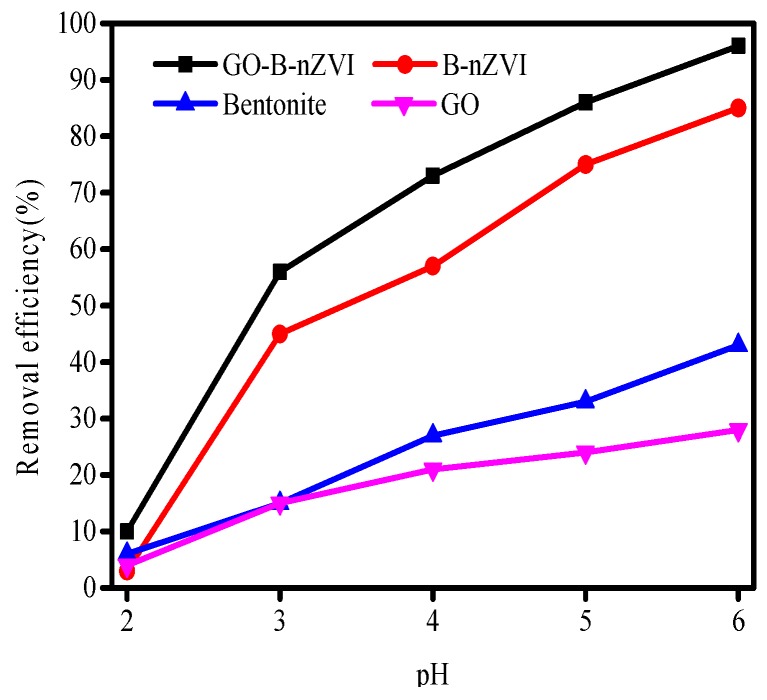
Removal efficiency of Cu(II) by bentonite, GO, B-nZVI, and GO-B-nZVI with pH (Temperature = 18 °C; adsorbent dose = 1 g·L^−1^; concentration of Cu(II) = 80 mg·L^−1^; adsorption time = 24 h).

The pH of the solution is among the most critical factors that affect the removal efficiency of Cu(II) for both the speciation of Cu(II) in solution, and the speciation of the surface of the adsorbent in contact with the solution. The effect of pH on adsorption efficiency is shown in Figure 8. Care was taken to not alter the initial concentrations of the Cu(II), with a maximum increase of 0.01 mL in volume due to pH change. Removal efficiency of Cu(II) by bentonite, GO, B-nZVI, and GO-B-nZVI increased with the change in pH from 2 to 6. The absorption of Cu(II) depends largely on pH, with stronger sorption of cations at high pH, and stronger sorption of anions at low pH [35]. The removal efficiency of Cu(II) was extremely low, only 3% to 10% of Cu(II) had been removed by the four materials when the pH was 2. One main factor was that fewer surface active sites of adsorbents could be accessible to Cu(II) because of the competition between Cu(II) and (H^+^) at low pH. In acidic environments, iron particles can be corroded by (H^+^), resulting in fewer iron particles reacting with Cu(II). When the pH value of the solution was 5, the removal efficiency of Cu(II) by GO-B-nZVI reached a relatively high value (86%).

**Figure 9:**
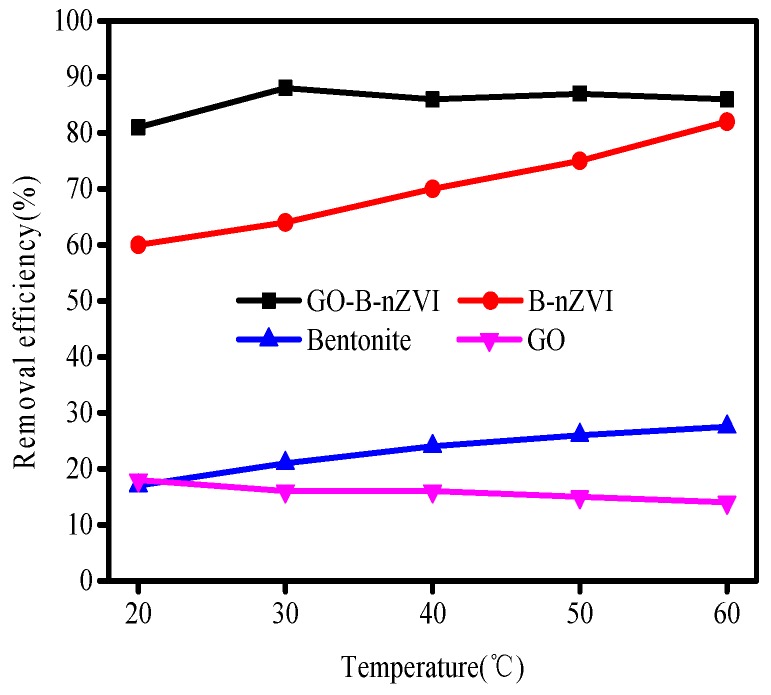
Removal efficiency of Cu(II) by bentonite, GO, B-nZVI, and GO-B-nZVI with time (Adsorbent dose = 1 g·L^−1^; pH = 5 ± 0.3; concentration of Cu(II) = 80 mg·L^−1^; adsorption time = 4 h).

To assess the effect of different temperatures, batch experiments were conducted at 20, 30, 40, 50, and 60 °C. The results showed that 82% Cu(II) was removed by B-nZVI at 60 °C while only 60% Cu(II) was removed at 20 °C in 24 h (Figure 9). Thus, the temperature had a positive influence on the reduction of Cu(II) by B-nZVI. The removal efficiency of Cu(II) by GO-B-nZVI increased from 81% to 88%, as the temperature increased from 20 °C to 30 °C. However, when the temperature exceeded 30 °C, the removal efficiency of Cu(II) did not change much, with a slight fluctuation as temperature increased. At different temperatures, GO-B-nZVI showed better removal efficiency of Cu(II) than that of B-nZVI, indicating that adding GO into the solution during the process of synthesizing the material could improve the performance of the nZVI.

### 3.3. Adsorption Kinetics and Adsorption Isotherms

In order to explore the adsorption behavior of graphene oxide/bentonite decorated nano-iron, two kinds of pseudo-first-order and second-order kinetic models were used to study the adsorption kinetics of the synthesized materials:

(3) $$\ln(q_{e}-q_{t})=\ln(q_{e})-k_{1}t$$

(4) $$\frac{t}{q_{t}}=\frac{1}{k_{2}q_{e}{}^{2}}+\frac{t}{q_{e}}$$

**Figure 10:**
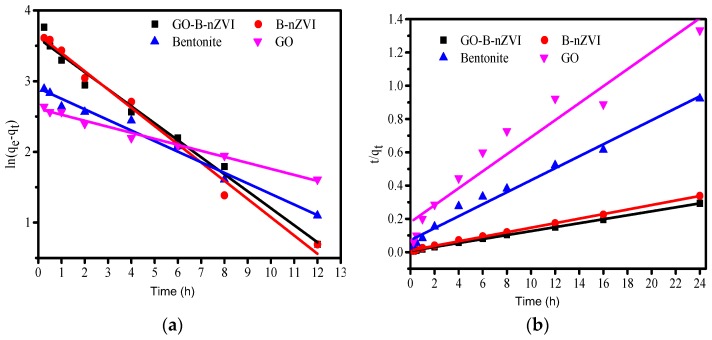
The pseudo first-order kinetic curve: (**a**) and pseudo-second-order kinetic and (**b**) curve of bentonite, GO, B-nZVI, and GO-B-nZVI for Cu(II) absorption.

Equations (3) and (4) are pseudo-first-order and pseudo-second-order kinetic equations, respectively, where q_e_ and q_t_ correspond to the amounts of adsorbed Cu(II) at equilibrium and time t (expressed in (mg·g^−1^)), respectively. The k_1_ and k_2_ are constants of the pseudo-first-order and pseudo-second-order adsorption rate [36,37]. The pseudo-first-order and pseudo-second-order kinetic model of the relevant parameters can be obtained according to the above equation fitting, as shown in Table 3. The k_1_ and k_2_ were likewise obtained by the fitting curve (Figure 10). By comparing the fitting results of the two models, it was found that the pseudo-second-order kinetics fit better for B-nZVI and GO-B-nZVI. The goodness of fit (R^2^) for B-nZVI and GO-B-nZVI were 0.999 and 0.999 when fitting with the pseudo-second-order kinetic model, which was better than that of the pseudo-first-order model (0.988 and 0.983). Furthermore, the equilibrium adsorption capacity (q_e,exp_/(mg·g^−1^)) of B-nZVI and GO-B-nZVI were 71 and 82 mg g^−1^ from the experiment, respectively. According to the pseudo-second-order kinetic model, q_e,cal_ (73.37 mg·g^−1^ and 83.89 mg·g^−1^) was closer to the experimental value q_e,exp_ when compared with the pseudo-first-order kinetic model (38.71 and 36.86). Therefore, the synthetic materials adsorbed Cu(II) more in line with the pseudo-second-order kinetic model.

Adsorption of copper ions by composite materials is a dynamic equilibrium process. In order to study the adsorption process of composite materials, we used the most commonly used chemical sorption behavior of the Langmuir and Freundlich isothermal model, as shown in Equations (5) and (6):

(5) $$\frac{c_{e}}{q_{e}}=\frac{1}{K_{L}q_{m}}+\frac{c_{e}}{q_{m}}$$

(6) $$\ln(q_{e})=\ln(K_{F})+\frac{1}{n}\ln(c_{e})$$

where q_m_ is the maximum adsorption capacity, mg·g^−1^; K_L_ is the constant related to adsorption equilibrium; K_F_ is the adsorption equilibrium constant of Freundlich; n is the constant of Freundlich model [38,39]. The Langmuir isothermal equation and Freundlich isothermal equation were used to fit the data of copper ions adsorbed by bentonite, GO, B-nZVI, and GO-B-nZVI, and the results are shown in Table 4.

**Figure 11:**
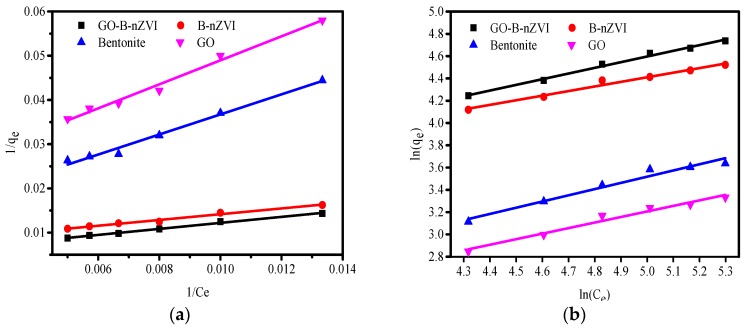
The Langmuir fit and Freundlich fit curve of bentonite, GO, B-nZVI, and GO-B-nZVI for copper ions absorption: (**a**) Langmuir model; (**b**) Freundlich model.

The correlation coefficient (R^2^) of the Langmuir model fit better than that of the Freundlich, indicating that the adsorption of copper ions by bentonite, GO, B-nZVI, and GO-B-nZVI was more in line with the Langmuir model, as shown in Figure 11. From the fitting curve, the values of q_m_ can be calculated. From the Langmuir model, the q_m_ of B-nZVI and GO-B-nZVI were 130.7 mg·g^−1^ and 184.5 mg·g^−1^, respectively, as shown in Table 4.

## 4. Conclusions

The study investigated the applicability of nanoscale zero-valent iron decorated on bentonite/graphene oxide (GO-B-nZVI) as an adsorbent to remove copper ions in aqueous solutions. It was clear that the agglomeration of nZVI could be eliminated and the successful attachment of nZVI to the bentonite and graphene oxide surface was confirmed. The addition of graphene oxide can improve the removal effect of copper ions when compared with nanoscale iron decorated on bentonite. The removal efficiency of copper ions by GO-B-nZVI increased first and then fluctuated slightly with the increase of temperature, indicating that GO-B-nZVI was insensitive to temperature change and had a high removal efficiency of copper ions at different temperatures in the experiment. GO-B-nZVI showed an increased removal efficiency of copper ions with the increase of pH under a certain pH range. A proposed reaction mechanism for copper ion reduction by GO-B-nZVI was mainly through the reduction capability of nZVI. In further research, the actual applicability of GO-B-nZVI remediation of heavy metals in soil will be studied.

## Figures and Tables

**Table 1:** Chemical composition of the bentonite.

SiO_2_	Al_2_O_3_	Fe_2_O_3_	TiO_2_	CaO	MgO	K_2_O	Na_2_O
61.62	14.18	2.45	0.4	1.77	2.33	2.13	1.1

**Table 2:** The BET area of bentonite, GO, B-nZVI, and GO-B-nZVI.

Samples	Surface Area (m^2^·g^−1^)	Average Pore Diameter (nm)
Bentonite	32.4	10.57
GO	53.80	18.75
B-nZVI	42.88	11.56
GO-B-nZVI	47.32	12.06

**Table 3:** Pseudo-first-order and pseudo-second-order kinetic model of the relevant parameters.

Absorbents	q_e,exp_/(mg·g^−1^)	Pseudo-First-Order Kinetic Model			Pseudo-Second-Order Kinetic Model		
		k_1_	q_e,cal_/(mg·g^−1^)	R^2^	k_2_·h^−1^	q_e,cal_/(mg·g^−1^)	R^2^
bentonite	26	0.1496	18.18	0.980	0.0207	27.74	0.982
GO	18	0.0851	13.61	0.985	0.0172	19.56	0.928
B-nZVI	71	0.2580	38.71	0.988	0.0190	73.37	0.999
GO-B-nZVI	82	0.2404	36.86	0.983	0.0211	83.89	0.999

**Table 4:** Fit data of copper ions adsorbed by bentonite, GO, B-nZVI, and GO-B-nZVI by the Langmuir isothermal equation and Freundlich isothermal equation.

Absorbents	Langmuir			Freundlich		
	q_m_/(mg·g^−1^)	K_L_/(L·mg^−1^)	R^2^	K_F_	n	R^2^
Bentonite	71.2	0.0062	0.985	2.0813	1.795	0.961
GO	45.9	0.0080	0.985	2.046	2.008	0.967
B-nZVI	130.7	0.0117	0.984	10.465	2.427	0.976
GO-B-nZVI	184.5	0.0080	0.993	7.766	1.965	0.996
